# Small intestinal mucosal injury and its risk factors in patients with gastrointestinal cancer who developed complicated fluoropyrimidine-induced diarrhea

**DOI:** 10.1186/s12876-020-01507-5

**Published:** 2020-10-27

**Authors:** Miho Sakumura, Takayuki Ando, Ayumu Hosokawa, Takahiko Nakajima, Iori Motoo, Hiroshi Mihara, Akira Ueda, Shinya Kajiura, Sohachi Nanjo, Haruka Fujinami, Kohei Ogawa, Ichiro Yasuda

**Affiliations:** 1 The Third Department of Internal Medicine, Faculty of Medicine, University of Toyama, 2630 Sugitani, Toyama, 930-0194 Japan; 2 Department of Diagnostic Pathology, Graduate School of Medicine and Pharmaceutical Sciences, University of Toyama, Toyama, Japan

**Keywords:** Mucosal injury, Gastrointestinal cancer, Chemotherapy, Fluoropyrimidine, Chemotherapy-induced diarrhea

## Abstract

**Background:**

Diarrhea is a common adverse event of fluoropyrimidine-based chemotherapy. However, limited data are available on the frequency and risk factors of complicated chemotherapy-induced diarrhea (CID) and small intestinal mucosal damage. In this current study, we aimed to determine the incidence of complicated CID and mucosal injury among patients with complicated CID receiving fluoropyrimidine via small bowel capsule endoscopy (CE) and determined baseline risk factors associated with complicated CID.

**Methods:**

In total, 536 patients with advanced or recurrent gastrointestinal cancer who received fluoropyrimidine-based chemotherapy were retrospectively analyzed. Diarrhea was evaluated using the Common Terminology Criteria for Adverse Events version 4. Complicated CID was defined according to the American Society of Clinical Oncology guidelines. To evaluate small intestinal mucosal injury in patients with complicated CID, CE was performed. Multivariate analysis was performed to identify risk factors for complicated CID.

**Results:**

Total number of 32 (6%) patients developed complicated CID. Complicating symptoms were noted in 25 (78%) patients, with cramping, vomiting, and sepsis being observed in 15 (60%), 8 (32%), and 3 (12%) patients, respectively. Among the 13 patients who underwent CE, 11 (85%) showed abnormal findings. Multivariate analysis revealed that oral fluoropyrimidine administration was a risk factor for complicated CID (odds ratio 2.95; 95% confidence interval 1.06–8.19).

**Conclusions:**

Despite the relatively low incidence of complicated CID, mucosal injury of small intestine was common in patients with complicated fluoropyrimidine-induced diarrhea and oral fluoropyrimidine was an independent risk factor.

## Background

Prospective clinical trials and meta-analyses in the 1990s demonstrated that chemotherapy had greater survival benefit than that of the best supportive care [1]. Cytotoxic chemotherapy, especially that involving use of fluoropyrimidines, prolongs survival and has been widely used in patients with various malignancies, including gastrointestinal, breast, and head and neck cancers. Recently, oral chemotherapy involving the use of fluoropyrimidines (capecitabine and S-1) has provided new perspectives for the treatment of gastrointestinal cancer owing to its greater simplicity and convenience than conventional chemotherapy involving 5-fluorouracil (5-FU) [2].

Nonetheless, cytotoxic side effects are serious issues hindering the clinical application of beneficial therapies. Common clinical toxicities of fluoropyrimidines result from the inhibition of rapidly dividing cells, such as bone marrow hematopoietic and gastrointestinal epithelial cells, causing cytopenia and diarrhea, respectively. Studies have shown that chemotherapy-induced diarrhea (CID), a common non-hematological toxicity, can be predicted based on dose, schedule, and administration route [3, 4]. Severe diarrhea has been noted in 8–37% of patients receiving fluoropyrimidines owing to lower gastrointestinal tract toxicity; diarrhea tolerance among patients within and outside the United States (US) has been found to differ [5]. Particularly, the incidence of CID among Japanese patients has been considered to be low, with studies reporting a frequency of 1–8% of grade 3–4 diarrhea [2, 6]. However, it can be severe in some patients, especially when sepsis occurs [7, 8].

Appropriate treatment for CID is important considering that it would allow patients to continue chemotherapy for cancer, leading to a better prognosis. Accordingly, only loperamide, octreotide, and opium tincture have been recommended in the updated treatment guidelines by the consensus conference on the management of CID [9]. From the perspective of treating CID, diarrhea can be classified as either uncomplicated or complicated [9]. Loperamide, which functions by decreasing intestinal motility, has been widely used as the primary treatment for CID, with additional aggressive management, including administration of antibiotics or octreotide, being recommended for complicated CID. However, details regarding aggressive management and its impact on patients have been limited. Furthermore, while evaluation of intestinal mucosal injury among patients with CID might be important for determining the treatment strategy, only a few reports have investigated the incidence or severity of small intestinal mucosal injury and the incidence of CID accompanied by complications.

The present study aimed to determine the incidence and risk factors of complicated CID and mucosal injury among patients receiving fluoropyrimidine using small bowel capsule endoscopy (CE).

## Methods

### Patients

Patients with advanced or recurrent gastrointestinal cancer who underwent fluoropyrimidine-based chemotherapy at Toyama University hospital between April 2006 and June 2017 were retrospectively analyzed. Clinical assessments were repeated every 2 or 3 weeks during chemotherapy, while information was retrospectively collected from medical records. This study was approved by the Institutional Review Board (No. 26-136), and all patients signed an informed consent for chemotherapy and capsule endoscopy.

### Treatment regimens and schedule

Chemotherapy regimens in clinical practice or within the context of a clinical trial were selected individually. The dosage and schedules of most chemotherapy regimens were based on previously reported recommendations [10, 11]. Treatment was continued until disease progression, occurrence of unacceptable toxicity, or patient refusal to continue therapy despite appropriate dose reduction.

### Evaluation and management of diarrhea

Diarrhea severity was classified according to the Common Terminology Criteria for Adverse Events (CTCAE) version 4. This includes: Grade 1, an increase of < 4 stools per day over baseline or a mild increase in ostomy output compared to baseline; Grade 2, an increase of 4–6 stools per day over baseline or a moderate increase in ostomy output compared to baseline associated with limited impact on activities of daily living (ADL); Grade 3, an increase of ≥ 7 stools per day over baseline requiring hospitalization or a severe increase in ostomy output compared to baseline associated with significant limitations with respect to self-care ADL; Grade 4, life-threatening condition requiring urgent intervention; Grade 5, death. Complicated diarrhea was defined according to the following American Society of Clinical Oncology guidelines: CTCAE grade 3 or 4 diarrhea or grade 1 or 2 diarrhea with one or more additional signs or symptoms, including cramping, nausea/vomiting (grade 2 or more), decreased performance status, fever, sepsis, neutropenia, frank bleeding, and dehydration [9]. Uncomplicated diarrhea was defined as grade 1 or 2 diarrhea with no complicating symptoms. Cramping associated with CID was defined as any case in which this symptom developed together with CID.

All patients diagnosed with complicated diarrhea were admitted to the hospital, and their chemotherapy was discontinued. Abdominal computed tomography (CT) scan was performed as needed, and bowel wall thickening was defined as demonstration of the bowel wall of more than 4 mm in thickness and over more than 30 mm in length.[12]. To distinguish between infectious and CID diarrhea, stool cultures (for *Clostridium difficile*, *Escherichia coli*, and other infectious agents that cause colitis), complete blood count, electrolyte panel, and CT scans were performed in patients who developed complicated diarrhea. Intravenous fluid and antibiotics were administered until all symptoms had resolved.

### Small bowel CE procedure and evaluation

Patients with complicated CID underwent CE when their condition improved to CTCAE grade 1, because the risk of capsule retention should be reduced [13]. Additionally, all patients who underwent CE satisfied the following criteria: (1) without massive ascites or severe peritoneal dissemination; (2) being able to continue chemotherapy; and (3) capable of oral intake. Furthermore, depending on each patient’s condition, a patency capsule was used to confirm intestinal patency according to the operator’s discretion.

Each patient was instructed not to consume solid food after 8 PM on the day before the procedure. CE studies were performed using the Pillcam™ SB2, SB3 system (Medtronic, Dublin, Ireland) or Endo Capsule™ system (Olympus, Tokyo, Japan). The monitoring period was approximately 10 h, corresponding to the battery life of the device. Two operators classified abnormalities observed in the video as red spots, erosions, and ulcers [14]. Red spots were predominantly distinguished from angiectasia based on size. Mucosal erosions and ulcers were classified according to the size of the small bowel mucosal breaks considering that ulcers, by definition, require a degree of penetration and that evaluating lesion depth based on the angle of the image taken by the capsule was often impossible [15]. When any of the findings were observed in more than two locations, they were scored as abnormal.

### Statistical analysis

The incidence of diarrhea and the number of days from initiation of fluoropyrimidine-based chemotherapy to the onset of complicated CID were investigated. Additionally, time-to-event curves were calculated using the Kaplan–Meier method and compared using the log-rank test. Logistic regression models were used to calculate odds ratios (ORs) and 95% confidence intervals (CIs) to identify which clinical factors influenced complicated CID. All statistical analyses were performed using EZR (Saitama Medical Center, Jichi Medical University, Saitama, Japan), a graphical user interface for R (The R Foundation for Statistical Computing, Vienna, Austria). More precisely, it is a modified version of R commander designed to add statistical functions frequently used in biostatistics. A *p* value of < 0.05 was considered to indicate statistical significance.

## Results

### Patients characteristics

The present study included 536 patients (256 with gastric cancer and 280 with colorectal cancer), the clinical characteristics of whom are presented in Table 1. The most common types of fluoropyrimidines administered were S-1 (70%) for gastric cancer and 5-FU (72%) for colorectal cancer. Both groups had low frequency of capecitabine use. The chemotherapy regimen consisted of fluoropyrimidine alone and combination chemotherapy in 77 (14%) and 459 (86%) patients, respectively, while molecular-targeted drug therapy consisted of fluoropyrimidine and cisplatin plus trastuzumab in 35 patients with HER2-positive gastric cancer. Patients with colorectal cancer received molecular-targeted drugs in combination with fluoropyrimidine and oxaliplatin or irinotecan. Bevacizumab and cetuximab/panitumumab were administered in 25 and 116 patients, respectively.

**Table 1:** Patient characteristics

	Total	Gastric cancer (n = 256)	Colorectal cancer (n = 280)
Sex			
Male/female	357/179	189/67	168/112
Age			
Median (range)	66 (11–87)	66 (22–87)	66 (11–86)
ECOG PS			
0–1/≥ 2	450/86	204/52	246/34
Stage			
Advanced	433 (81%)	225 (88%)	208 (74%)
Postoperative recurrence	103 (19%)	31 (12%)	72 (26%)
Metastatic sites			
Lymph node	279 (52%)	184 (72%)	95 (34%)
Liver	253 (47%)	97 (38%)	156 (56%)
Peritoneum	131 (24%)	101 (39%)	60 (21%)
Number of metastatic sites			
0–1/≥ 2	233/303	113/143	120/160
Fluoropyrimidine type			
5-FU	262 (49%)	43 (17%)	219 (72%)
S-1	182 (34%)	178 (70%)	4 (1%)
Capecitabine	85 (16%)	32 (12%)	53 (19%)
Others	7 (1%)	3 (1%)	4 (1%)
Molecular-targeted drugs			
Trastuzumab	35	35	0
Cetuximab/panitumumab	25	0	25
Bevacizumab	116	0	116
Chemotherapy regimens			
Fluoropyrimidine alone	69 (13%)	55 (21%)	14 (5%)
Combination chemotherapy	467 (87%)	201 (79%)	266 (95%)
Platinum	389	161	228
Irinotecan	29	0	29
Taxane	24	24	0
Others	17	16	1

*ECOG PS* Eastern Cooperative Oncology Group performance status, *5-FU* 5-fluorouracil

### Complicated diarrhea

**Fig. 1:**
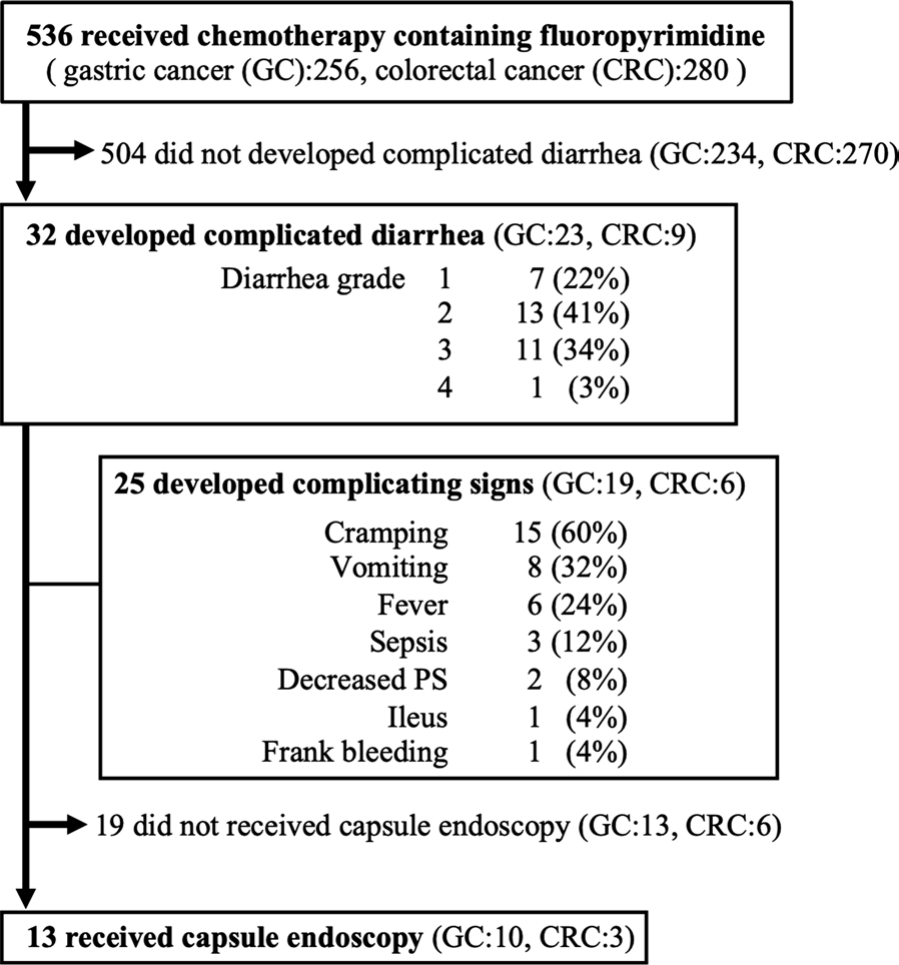
Flow diagram of data collection and analysis. *GC* gastric cancer, *CRC* colorectal cancer

**Fig. 2:**
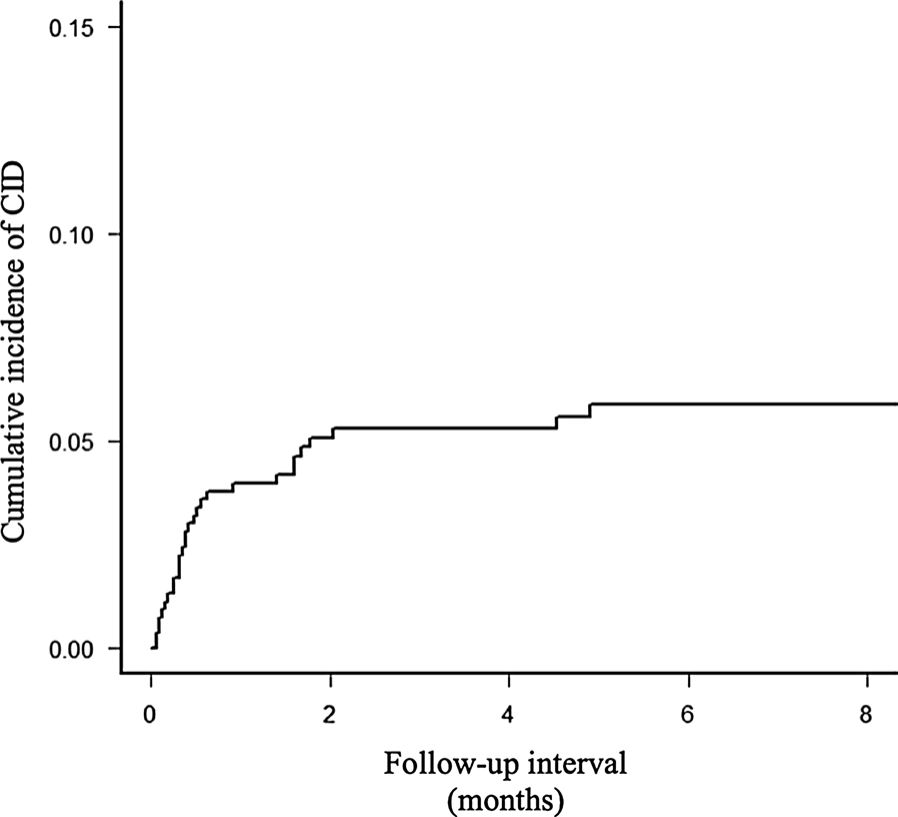
Cumulative incidence of complicated chemotherapy-induced diarrhea (CID). Most patients showed CID within a month after administration of chemotherapy

Among 536 patients, diarrhea with grade 1 or 2 and grade 3 or 4 were observed in 174 (32%) and 10 (2%) patients, respectively. A total of 32 (6%) patients developed complicated CID, among whom 23 and 9 had gastric and colorectal cancer, respectively. Most of the patients developed the condition within a month (Figs. 1, 2). Complicating symptoms were noted in 78% (25/32) patients, 76% (19/25) of whom were with oral fluoropyrimidine and 24% (6/25) of whom were with infusional fluoropyrimidine. Accordingly, cramping, vomiting, fever and sepsis were observed in 15 (60%), 8 (32%), 6 (24%), and 3 (12%) patients.

Among the 32 patients with complicated CID, 6% (2/32) and 94% (30/32) developed the condition after receiving single-agent chemotherapy and combination chemotherapy, respectively. All patients, except one who died after developing grade 4 neutropenia and sepsis, recovered from complicated CID through infusion and antibiotic therapy. The median duration to recovery from complicated CID to grade 1 diarrhea was 6 days (range 1–59 days).

### Small bowel findings of CT scan and CE

Among 32 patients who developed complicated CID, 13 patients underwent CE (10 gastric and 3 colon cancer; 11 oral fluoropyrimidine and 2 infusional 5-FU based chemotherapy). Table 2 summarizes the characteristics of patients with complicated CID who underwent CE. 59% (19/32) patients did not undergo CE because of following reasons. Eleven patients had the risk of retention: due to primary tumours in 8, peritoneal dissemination in 2, and massive ascites in 1. Seven patients refused the examination despite extensive explanation. Another patient was unable to undergo CE due to the severity of the underlying medical condition. Six patients received a patency capsule, and all of them were confirmed to have intestinal patency. The mean duration between the onset of complicated CID and small bowel CE was 9 days (range 2–21 days). Among the 13 patients who underwent CE, the capsule passed through their small intestine within the scheduled timeframe in 10 patients. Capsule excretion was ultimately confirmed in all 13 patients with no adverse events including capsule retention.

**Table 2:** Characteristics of patients with complicated chemotherapy-induced diarrhea who underwent capsule endoscopy

No	Cancer	PS	Treatment	Cycle	Diarrhea grade	Complicating signs	Neutrophil count*	Wall thickening of small intestine	Schedule time**	Endoscopic findings		
										Redness	Erosion	Ulcer
1	Gastric	1	Capecitabine Cisplatin Trastuzumab	1	2	Cramping, sepsis	4070	+	14	+	+	–
2	Gastric	1	Capecitabine Cisplatin Trastuzumab	3	2	sepsis	5330	+	17	+	+	–
3	Gastric	1	S-1 Docetaxel Cislatin	1	1	Cramping, fever	6600	–	19	+	+	–
4	Gastric	2	S-1 Oxaliplatin	1	3	Cramping fever	3100	N/A	11	+	+	–
5	Gastric	1	S-1 Cisplatin	1	1	Decreased PS fever	2650	+	2	+	+	+
6	Gastric	1	Capecitabine Oxaliplatin	1	3	Cramping fever	4290	–	9	+	–	–
7	Gastric	1	5-FU	4	3	Cramping	4520	+	8	+	+	+
8	Gastric	1	S-1 Oxaliplatin	1	2	Cramping	3210	–	2	–	–	–
9	Gastric	1	S-1 Cisplatin	5	3	–	6570	–	7	+	–	–
10	Gastric	0	S-1	8	3	Vomiting	2880	–	5	–	–	–
11	Colon	2	5-FU Oxaliplatin	1	1	Cramping ileus	2990	+	3	+	+	–
12	Colon	1	S-1 Irinotecan Bevacizumab	1	2	Cramping vomiting	3490	+	13	+	–	–
13	Colon	1	Capecitabine Oxaliplatin	1	3	Cramping	2170	+	21	+	+	+

*PS* performance status, *5-FU* 5-fluorouracil, *N/A* not available

^*^cells/mm^3^, **Number of days after the end of chemotherapy courses and capsule endoscopy

**Fig. 3:**
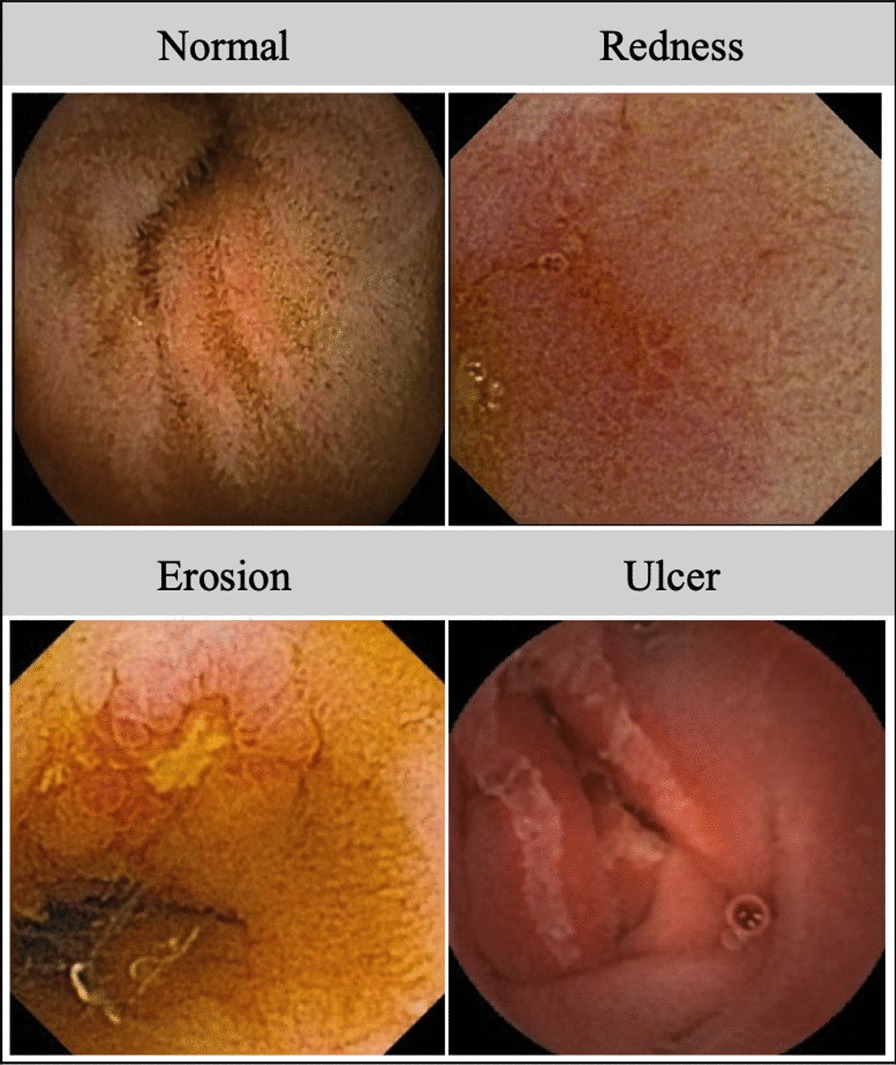
Representative capsule endoscopy images of small intestinal mucosal injuries. Normal finding is observed in patients with fluoropyrimidine-based chemotherapy without diarrhea. Redness is a reddened fold, erosion is a white spot surrounded by a halo, and ulcer is a depression with a white coating

**Fig. 4:**
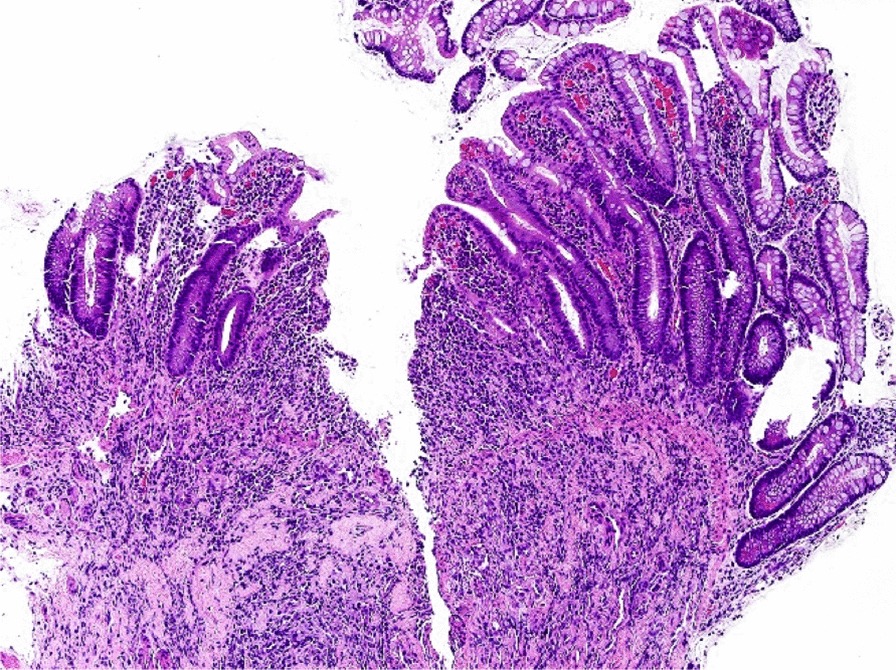
Microscopic view of the biopsy specimen obtained from ileal ulcer. Acute and chronic inflammatory infiltrate is observed within and near the ulceration

Among the 13 patients who underwent CE, CT scans were performed in 12 at the onset of complicated CID. Increased thickness of the wall of the small intestines was identified in 7 patient cases; however, no abnormalities associated with the large intestines were detected. Moreover, of the four patients with mucosal injury of the small intestine, 2 patients (#2 and #6) underwent esophagogastroduodenoscopy and the other 2 patients (#5 and #11) underwent colonoscopy. No abnormal findings were detected in either of these studies except for previously-identified primary tumors or mucosal injury of small intestine. Among those who underwent CE, abnormal findings were identified in 85% (11/13) patients; multiple red spots, erosions, and ulcers were noted in 91% (10/11), 64% (7/11), and 27% (3/11) patients, respectively (Fig. 3). Of the 11 patients who presented with abnormal findings, mucosal lesions were detected in the ileum in six patients (#1, #3, #7, #9, #12, and #13), in the jejunum in four patients (#2, #4, #5, and #6), and in entire small intestine in one patient (#11). Colonoscopy was performed in patient #5 on day 13 after CE (see Table 2), which revealed sparsely-distributed small ulcers and erosions that were amenable for biopsy. Histology of an ileal biopsy specimen revealed ulceration with granulation tissue with acute and chronic inflammatory changes (Fig. 4). Of the 536 patients enrolled in this study, 349 patients (65%) experienced no diarrhea. Capsule endoscopy (CE) was performed in five of these patients (2 diagnosed with gastric cancer and 3 with colon cancer; 3 had undergone treatment with oral fluoropyrimidine and 2 with intravenous 5-FU). No abnormal findings were detected in any of the patients in this cohort.

### Clinical factors associated with the incidence of complicated CID

Univariate and multivariate analyses were performed to identify clinical or treatment factors associated with the incidence of complicated CID (Table 3) given that small intestinal mucosal injury was observed in most of the patients with complicated CID. Univariate analysis involving the entire patient cohort indicated that gastric cancer (*p* = 0.01) and oral fluoropyrimidine (*p* = 0.003) were significantly associated with the incidence of complicated CID, whereas sex, age, performance status, primary site resection, number of metastatic sites; however, any combination of drugs including platinum, irinotecan, taxane, trastuzumab, cetuximab or panitumumab, and bevacizumab were not. Multivariate analysis revealed that oral fluoropyrimidine (OR 2.95; 95% CI 1.06–8.19) was independent risk factor associated with the incidence of complicated CID.

**Table 3:** Univariate and multivariate analyses of factors associated with complicated chemotherapy-induced diarrhea

Factor	Univariate analysis			Multivariate analysis		
	OR	95% CI	*p*	OR	95% CI	*p*
Cancer sites, stomach (/colorectum)	2.53	1.17–5.47	0.01	1.38	0.55–3.47	0.48
Sex, female (/male)	1.39	0.67–2.88	0.37			
Age, ≥ 70 (/< 70)	1.26	0.58–2.72	0.54			
PS, 0–1 (/ ≥ 2)	1.03	0.38–2.76	0.94			
Resection of primary sites, no (/yes)	2.42	0.72–8.11	0.15			
Number of metastatic sites, 0–1 (≥ 2)	1.75	0.85–3.59	0.12			
Fluoropyrimidine type*, oral (/infusional)	3.59	1.52–8.47	0.003	2.95	1.06–8.19	0.03
Combination chemotherapy, yes (/no)	0.78	0.29–2.11	0.63			
Platinum, yes (/no)	0.92	0.41–2.05	0.84			
Irinotecan, yes (/no)	2.52	0.82–7.71	0.10			
Taxane, yes (/no)	0.32	0.04–2.46	0.27			
Trastuzumab, yes (/no)	0.95	0.21–4.15	0.94			
Cetuximab/panitumumab, yes (/no)	0.43	0.05–3.26	0.41			
Bevacizumab, yes (/no)	0.65	0.24–1.74	0.39			

*OR* odds ratio, *CI* confidence interval, *PS* performance status

^*^Oral fluoropyrimidine includes S-1 or capecitabine, and intravenous fluoropyrimidine includes 5-fluorouracil

The incidence of diarrhea of all grades was 38% (101/274) and 31% (82/262) among the patients treated with oral fluoropyrimidine and intravenous 5-FU, respectively; this difference did not reach statistical significance (*p* = 0.09). By contrast, the incidence of complicated CID in the patients treated with oral fluoropyrimidine and intravenous 5-FU was 9% (25/276) and 3% (7/260), respectively, a finding that was highly significant (*p* = 0.001).

## Discussion

The present study carefully evaluated the small intestinal mucosa of patients with gastrointestinal cancer who developed complicated CID using CE. After chemotherapy discontinuation, almost all patients recovered from the condition, which had developed within a month. CE revealed that large proportion of patients with complicated CID developed multiple mucosal injuries even after the improvement to CTCAE grade 1 diarrhea. Furthermore, oral fluoropyrimidine was the independent risk factor associated with occurrence of complicated CID.

The consensus guidelines of CID do not provide sufficient evidence for its diagnosis and treatment, although it has been suggested the need for intensive evaluation and aggressive treatment, including stool work up, complete blood count, electric profile, intravenous fluid administration and antibiotic therapy, among patients with complicated CID [9, 16]. Moreover, it can be difficult to differentiate between infectious and non-infectious diarrhea that develops in patients undergoing chemotherapy. One of the most critical elements of the differential diagnosis is neutropenic enterocolitis, which is a condition associated with neutrophil counts < 500/L, fever, abdominal pain, and bowel wall thickening [12, 17]. In this study, only one out of the 32 patients who experienced complicated CID developed severe neutropenia associated with diarrhea; this individual died without recovering from sepsis and neutropenia and CE was not performed. We have considered the possibility that this patient may have succumbed to neutropenic enterocolitis. However, none of the 13 patients who were evaluated with CE presented with neutropenia, including 3 patients who developed sepsis as a complication. In one patient with sepsis, blood culture revealed bacteremia with *Klebsiella pneumoniae*; this finding may have been the result of bacterial translocation from the gut due to small intestinal mucosal injury. Thus, given that patients with complicated CID are at significant risk for infection, aggressive treatment, including antibiotics and octreotide in addition to loperamide may be necessary [18]. Indeed, a recent randomized-controlled trial comparing octreotide with loperamide in 41 patients with 5-FU-induced diarrhea revealed that the patients treated with octreotide experienced superior control of diarrhea compared to those treated with loperamide (90.4%, [19/21] vs. 15.0% [3/20]; *p* < 0.05) [19].

While earlier studies conducted in mice models have established the microscopic features of gastrointestinal mucositis in 5-FU toxicity, recent reports have shown NF-κB and IL-4 to be critical mediators in this process [20–22]. In fact, ileal biopsy with colonoscopy in patients with 5-FU-induced diarrhea revealed marked acute and chronic inflammation, which might correspond with the pathophysiology of fluoropyrimidine in rodent models [8]. However, the extent and severity of this damage has yet to be studied in detail given that a considerable portion of the small intestine is beyond the reach of a colonoscope. Two recent studies evaluated the small intestinal mucosa of patients receiving chemotherapy [23, 24]. Accordingly, after performing CE in only those with diarrhea grades 0–2, both studies revealed that diarrhea grade was significantly correlated with the percentage of patients with intestinal mucosal injury. In contrast, the present study found a high incidence of mucosal injury regardless of diarrhea severity, perhaps because our endoscopic study targeted patients with complicated diarrhea, which includes both diarrhea severity and clinical factors. This finding suggests that complicating symptoms reflect chronic inflammation induced by fluoropyrimidine even among patients with grade 1 diarrhea. By contrast, no abnormal findings were detected in five patients undergoing fluoropyrimidine-based chemotherapy who did not develop diarrhea, although the patient cohorts were not background-matched. Therefore, these findings suggest fluoropyrimidine chemotherapy induced mucosal injury of small intestine, and assessing symptoms, such as fever, and cramping, are important for the management CID based on our endoscopic findings.

Recent studies have found that female sex, older age, and a normal body mass index were clinical factors predictive of fluoropyrimidine-induced diarrhea; however, none of them have been definitively established [25–27]. The present study showed that the aforementioned factors were not predictive of complicated CID. On the other hand, those receiving oral fluoropyrimidine chemotherapy had higher risk for CID compared to those receiving infusional 5-FU chemotherapy. A meta-analysis including 26 phase II and III trials on solid tumors revealed that S-1 had an OR of 1.03 (95% CI 0.87–1.22) for all grade diarrhea for compared with infusional 5-FU, and another network meta-analysis showed that the toxicity profiles of stomatitis and diarrhea did not differ between S-1 and capecitabine [28, 29]. In this study, the incidence of complicated CID was significantly higher in patients undergoing treatment with oral fluoropyrimidine compared with those who received intravenous 5-FU; however, the incidences of all grades of diarrhea were not significantly different. Indeed, maximum plasma concentrations and area under the curve of 5-FU during treatment with oral S-1 were significantly greater than the respective values associated with intravenous 5-FU; moreover, abdominal discomfort and cramping were identified as among the principal dose-limiting toxicities of S-1 in a phase I study [30, 31]. These abdominal symptoms were among those observed most frequently as therapeutic complications in this study. Additionally, genetics might also contribute to drug-specific toxic effects, with one study showing that dihydropyrimidine dehydrogenase (DPD) deficiency was associated with reduced clearance of fluoropyrimidines and prolonged exposure [32]. However, the most common genetic mutations are absent among Asians, while DPD testing has not been routinely recommended [10]. Therefore, the influence of genetics in this study, which included only Asian patients, appears to be relatively low, although DPD testing has not been performed.

Several limitations of the present study warrant consideration. First, CE had been performed during the recovery phase of CID; as such, the findings from endoscopy may be underestimated or inaccurate. However, we were unable to perform CE in patients experiencing diarrhea due to the increased risk of capsule retention. In this study, small intestinal thickness was detected by CT scan in 7 of 13 patients who underwent CE. These conditions may lead to gastrointestinal obstruction and capsule retention even if the patency capsule passes through intestine [13]. Nevertheless, mucosal injury had been noted in 85% of the patients who underwent CE, more patients might have shown abnormal endoscopic findings if the examination was performed during the acme phase of diarrhea. Second, the study included patients who received both combination chemotherapy and fluoropyrimidines alone. In addition, patients treated with irinotecan or cetuximab/panitumumab, which has been known to induce diarrhea, had been included, although these agents were not associated with complicated CID in univariate and multivariate analysis. Third, most patients developed complicated CID after their first cycle of each chemotherapy regimen, whereas others developed the condition after a few cycles. We believe that later-onset diarrhea could have been associated with factors other than chemotherapy, such as infection, which had not been identified by the stool culture.

## Conclusion

The mucosal injury of small intestine was common in patients with complicated fluoropyrimidine-induced diarrhea, although its incidence was relatively low. Moreover, oral fluoropyrimidine was an independent risk factor associated with complicated CID.

## Data Availability

The datasets during and/or analyzed during the current study are available from the corresponding author on reasonable request.

## Abbreviations

CE: Capsule endoscopy
CEST: Capsule endoscopy structured terminology
CI: Confidence intervals
CID: Chemotherapy-induced diarrhea
CTCAE: Common Terminology Criteria for Adverse Events
DPD: Dihydropyrimidine dehydrogenase
OR: Odds ratios
